# Imaging and modelling of poly(3-hydroxybutyrate) synthesis in *Paracoccus denitrificans*

**DOI:** 10.1186/s13568-021-01273-x

**Published:** 2021-08-09

**Authors:** Sergio Bordel, Rob J. M. van Spanning, Fernando Santos-Beneit

**Affiliations:** 1 Department of Chemical Engineering and Environmental Technology, School of Industrial Engineering, University of Valladolid, Dr. Mergelina, s/n, 47011 Valladolid, Spain; 2 Institute of Sustainable Processes, Dr. Mergelina s/n, 47011 Valladolid, Spain; 3 Department of Molecular Cell Biology, Faculty of Science, Vrije Universiteit Amsterdam, Amsterdam, The Netherlands

**Keywords:** Poly-3-hydroxybutyrate (PHB), Polyhydroxyalkanoates (PHA), Storage inclusions, Nile Red, LSCM, Metabolic modelling, Acetyl-CoA

## Abstract

Poly(3-hydroxybutyrate) (PHB) granule formation in *Paracoccus denitrificans* Pd1222 was investigated by laser scanning confocal microscopy (LSCM) and gas chromatography analysis. Cells that had been starved for 2 days were free of PHB granules but resynthesized them within 30 min of growth in fresh medium with succinate. In most cases, the granules were distributed randomly, although in some cases they appeared in a more organized pattern. The rates of growth and PHB accumulation were analyzed within the frame of a Genome-Scale Metabolic Model (GSMM) containing 781 metabolic genes, 1403 reactions and 1503 metabolites. The model was used to obtain quantitative predictions of biomass yields and PHB synthesis during aerobic growth on succinate as sole carbon and energy sources. The results revealed an initial fast stage of PHB accumulation, during which all of the acetyl-CoA originating from succinate was diverted to PHB production. The next stage was characterized by a tenfold lower PHB production rate and the simultaneous onset of exponential growth, during which acetyl-CoA was predominantly drained into the TCA cycle. Previous research has shown that PHB accumulation correlates with cytosolic acetyl-CoA concentration. It has also been shown that PHB accumulation is not transcriptionally regulated. Our results are consistent with the mentioned findings and suggest that, in absence of cell growth, most of the cellular acetyl-CoA is channeled to PHB synthesis, while during exponential growth, it is drained to the TCA cycle, causing a reduction of the cytosolic acetyl-CoA pool and a concomitant decrease of the synthesis of acetoacetyl-CoA (the precursor of PHB synthesis).

**Supplementary Information:**

The online version contains supplementary material available at 10.1186/s13568-021-01273-x.

## Key points

Modelling PHB synthesis, imaging the onset of PHB granules and their localization

## Introduction

Various microorganisms accumulate polyhydroxyalkanoates (PHAs) when carbon and energy sources are readily available. These polymers serve as reserves of carbon and energy to allow cell survival during starved conditions (Jendrossek and Handrick 2002). They have attracted industrial interest because they are completely biodegradable, non-toxic, biocompatible and naturally produced (Anderson and Dawes 1990; Dariš and Knez 2020). *Paracoccus denitrificans*, a gram-negative bacterium, accumulates poly(3-hydroxybutyrate) (PHB) as insoluble granules inside the cell (Ueda et al. 1992). PHB is the most prominent member of the PHAs and one of the best-studied bacterial storage polymers (Jendrossek 2009). Biosynthesis and biodegradation of PHB and other PHAs have been extensively investigated during the last three decades (Potter and Steinbüchel 2005; Jendrossek and Pfeiffer 2014). One exciting outcome of these studies was the finding that PHB granules are complexly organized subcellular structures and appear to be more than simple polymer inclusions (Jendrossek 2009). There are controversial results on the specific subcellular localization of the PHB granules in distinct bacterial models. Many studies show microscopy images with bacterial cells full of randomly localized PHB granules (Reinecke and Steinbüchel 2009), while other studies suggest a more organized pattern of PHB granules more or less in the middle of cells (Tian et al. 2005), or in the cell periphery near the cell poles (Jendrossek 2005). The aim of this work was twofold. Firstly, the investigation of PHB synthesis in *P. denitrificans* by both microscopy and biochemical analyses to study pattern formation of the granules. Secondly, the application of a functional Genome-Scale Metabolic Model (GSMM) of *P. denitrificans* Pd1222 to predict the metabolic flux distributions during the initial phase of fast PHB accumulation and the subsequent phase of exponential growth. The rationale was that GSMMs are comprehensive compilations of all metabolic reactions taking place in a particular cell, and as such can be used to predict theoretical yields of a bacterium during growth on different carbon and energy sources. As a result, we estimated the rates of biomass yield and PHB formations during growth on succinate. Consequently, in this study we investigated the formation of PHB granules and subcellular localization in *P. denitrificans*, a denitrifying model organism with a well-documented ability to accumulate PHB.

## Materials and methods

### Bacterial strains and growth conditions

*Paracoccus denitrificans* (Pd1222 Rif^R^, De Vries et al. 1989) was cultivated in batch cultures at 34 °C with either vigorous shaking 300 rpm or low shaking (150 rpm). Cultures were performed in a defined mineral salts medium (Harms et al. 1985) containing a trace solution and 25 mM succinate as the sole energy and carbon source. When required precultures from glycerol stocks were performed in LB supplemented with 50 μg ml^−1^ of rifampicin. Growth of *P. denitrificans* was measured spectrophotometrically (at 660 nm) using a SPECTROstar Nano spectrophotometer (BMG Labtech) and by quantifying dry weight biomass. For dry weight determination, three culture samples (2 ml) were washed twice with MilliQ water and dried for 5 days at 80 °C. When required, optical dense readings were used as a reproducible, high throughput method of growth phenotyping. Conversion of OD measurements to an estimated biomass concentration value was done as follow: Biomass (g L^−1^) = OD_660_ × 0.365 g L^−1^.

Initiation of PHB granule formation was performed by transfer of PHB-depleted cells to a fresh mineral salts medium with 25 mM succinate as the sole carbon and energy sources. For the preparation and imaging of spheroplasts, the *P. denitrificans* cells at an OD_660_ of about 1 were first incubated with 1 μM MTG for 20 min at 34 °C (to stain the PHB inclusions). Then, the stained cells were concentrated and washed in cold 100 mM Tris/HCl (pH 7.3), and finally resuspended in 100 mM Tris/HCl (pH 7.3) to a final OD_660_ of about 50. After that, 1 ml of the concentrated cells were treated with 50 µl of protease inhibitor + 10 µl of 200 mM EDTA + 1 ml of 200 mM Tris/HCl (pH 7.3)/1 M sucrose + 40 µl of lysozyme (50 mg.ml^−1^) during 30 min at room temperature. Finally, a mild osmotic shock was administered by the addition of 1 ml of ice-cold water. Spheroplasts were then centrifuged and resuspended in 100 mM Tris/HCl (pH 7.3) to a final OD_660_ of about 12 and then a drop of this dilution was imaged by LSCM as described in the section below. To image PHB granule formation at the desired time points, 1 ml of culture was incubated with 2 µM of Nile Red for 5 min at room temperature. Then, cells were washed with phosphate-buffered saline (PBS) and concentrated to a final OD_660_ of about 20 for microscopic analyses (see below).

### Fluorescent staining and laser scanning confocal microscopy (LSCM)

Cultures were incubated with the following dyes and concentrations: (1) MitoTraker Green FM (1 μM), (2) FM4-64 (1 μM), (3) Nile Red (1–2 μM), (4) Syto 80 (500 nM). Bacterial samples were taken at defined time points and were immediately stained by the addition of the corresponding dye at the desire concentration and incubated for at least 5 min at 34 ºC or room temperature. Cells were concentrated by centrifugation, washed once with PBS and normally re-suspended in PBS to a final OD_660_ ~ 20. Bacteria were immobilized on glass by mixing a drop of a concentrated bacterial suspension with a drop of a 1% low-melted agarose solution and immediately covered by a coverslip.

Cells were visualized with a BioRad Laser Scanning System Radiance 2000 confocal microscope. Blue-Argon Laser (488 nm) excited MTG and Green-Helium/Neon Laser (543 nm) excited the membrane dyes and DNA dyes used in this study. Images were converted from the Bio-Rad PIC to TIFF format using the public domain software ImageJ. Images were pseudo-coloured for enhanced visualization of differences in fluorescence intensity. The Blue-Argon Laser images were pseudo-coloured to green and the Helium/Neon images were pseudo-coloured to red. A 60 × oil objective was used to take the pictures and a 10 × extra zoom of the LaserSharp2000 program to magnify the size of the cells.

### Analytical procedures

The residual succinate concentration in the medium was measured by HPLC in 2 ml of filtered supernatant samples (0.22 μm) through an Alliance Waters HPLC equipped with an Aminex HPX-87H column (7.8 mm × 300 mm). The mobile phase was H_2_SO_4_ (25 mM), the column temperature was 75 °C and the eluent flow rate was 0.7 ml min^−1^. Instrument linearity was evaluated with succinate (ReagentPlus, 99%; Sigma-Aldrich) in the concentration range of 0.8–67.8 mM. The R^2^ obtained was 1.

Quantitative determination of PHB was carried out by adapting the method described in Zúñiga et al. (2011). Samples of 1.5 ml were centrifuged (14,000 rpm, 10 min) and the supernatant was discarded. Then, 1 ml of a solution of 1-propanol:HCl (80:20 *v/v*, 37% HCl *w/v*), 50 µl of the internal standard (benzoic acid) in 1-propanol (25 g L^−1^) and 2 ml of chloroform were added to the pellets and incubated for 4 h at 100 °C in a Thermoreaktot TR 300 (Merck KGaA, Darmstadt, Germany). After digestion, 1 ml of deionized water was added, and the suspension was vortexed. The organic phase was collected and filtered through 0.22 µm fibreglass filters. The propanly-ester of the butyric acid (the monomer constituting the PHB polymer) was measured in a 7820A GC coupled with a 5977E MSD (Agilent Technologies, Santa Clara, USA) and equipped with a DB-wax column (30 m × 250 μm × 0.25 μm). The detector and injector temperatures were maintained at 250 ºC. The oven temperature was initially maintained at 40 °C for 5 min, increased at 10 °C min^−1^ up to 200 °C (maintained at this temperature for 2 min) and then increased up to 240 °C at a rate of 5 °C min^−1^. Finally, PHB values were expressed as percentage dry cell weight based on the internal standard and the standard curve of Poly (3-hydroxybutyric acid) (Sigma-Aldrich®, USA).

### Genome-Scale Metabolic Model and FBA simulation

The genome of *P. denitrificans* Pd1222 was downloaded from GenBank (GCA_000203895.1). The genome was annotated using RAST (Overbeek et al. 2005), and a draft metabolic model was generated with SEED (Overbeek et al. 2014). The initial draft was manually curated (Bordel et al. 2019a), by checking gene annotations and making sure that genes annotated as enzymes are linked to the corresponding metabolic function, as reported in the database KEGG. Model manipulations and FBA simulations were carried out using the python library COBRApy (Ebrahim et al. 2013), version 0.9.1 with python 2.7.12. FBA simulations have been carried out by setting specific growth rates and specific PHB production rates, and minimizing the succinate uptake rate, The result of this simulation provides an estimation of all the metabolic reaction rates taking place in the cells.The model in SBML format (Paracoccus_denitrificans.xml) and the associated lists of metabolites (Paracoccus_metabolites.txt) and reactions (Paracoccus_reactions.txt) in tab-separated formats, Thave been deposited in https://github.com/SergioBordel/ModelParacoccus. The RAST annotation, both in Excel and GenBank formats, have been placed in the same repository.

## Results

### PHB granules formation by *P. denitrificans* cells using confocal microscopy

**Fig. 1:**
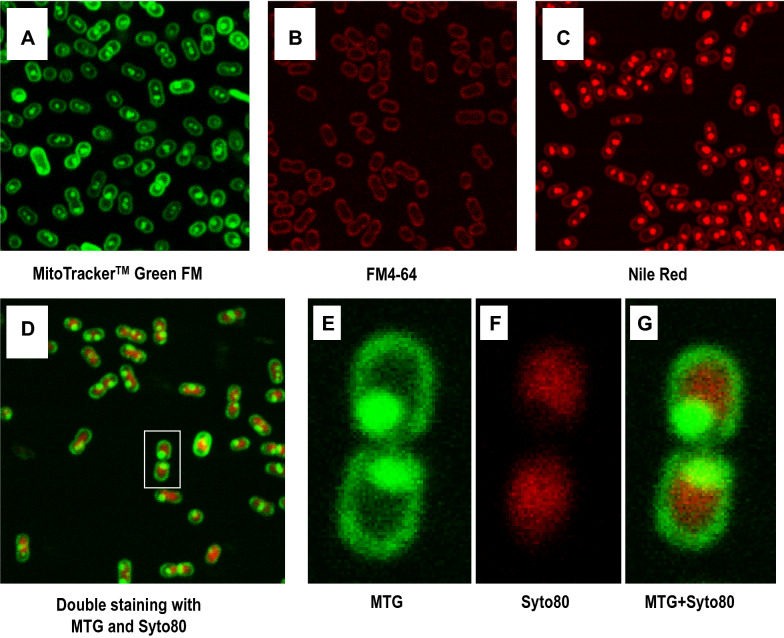
Staining of *P. denitrificans* cells with different fluorescent dyes. In all cases, cells were grown to an OD_660_ of about 0.75–1 and stained with: **A** MitoTracker™ Green FM (MTG) (1 µM); **B** FM4-64 (1 µM); **C** Nile Red (1 µM); **D** Double staining with MTG (1 µM) and Syto80 (500 nM). **E**–**G** Details of daughter cells from a recently divided mother cell. MTG image (**E**), Syto80 image (**F**), overlay (**G**). Cells were imaged using two different lasers (a blue-argon Laser, 488 nm, for excitation of MTG and a green-helium/neon laser, 543 nm, for excitation of Syto80)

The lipophilic core of PHB granules is covered by a monolayer of phospholipids (Steinbüchel et al. 1995); therefore only appropriate lipophilic dyes can be used to stain it (Vida and Emr 1995). PHB inclusions have been widely stained using Nile Red and a good linear correlation between the former and the PHB content has been well established in many previous works using different bacteria (Gorenflo et al. 1999; Spiekermann et al. 1999; Zuriani et al. 2013). *P. denitrificans* cells growing at the mid-exponential phase of growth were stained with different membrane dyes, including: MitoTracker™ Green FM (MTG), FM4-64 and Nile Red, and analyzed by LSCM. Figure 1 shows that both MTG and Nile Red (but not FM4-64) were able to stain the PHB granules. To our knowledge this the first time that staining of PHB inclusions is achieved using MTG (a dye normally used to stain mitochondria). No significant differences were observed between the staining patterns of Nile Red and MTG, except that the fluorescence intensity with Nile Red was stronger. The fluorescent images show that almost all of the cells growing at their exponential phase of growth contained PHB granules ranging from one to three in single cells and from two to six in dividing cells. Occasionally, we found cells with more structured and symmetric patterns of distribution of the PHB granules; see examples in Additional file 1: Figure S1A. Interestingly, this structured distribution of the PHB granules was also observed in spheroplasts of *P. denitrificans*; see examples in Additional file 1: Figure S1B.

We also carried out double-staining experiments of *P. denitrificans* cells growing at their mid-exponential phase of growth using MTG as membrane dye and Syto80 as DNA staining dye (see Fig. 1D–G). Figure 1D shows two daughter cells of a recently divided cell with PHB granules located at the poles and their DNA in the centre, indicative for mutual exclusion of these two types of molecules.

### PHB granules in *P. denitrificans* cells are synthesized de-novo within 30 min

**Fig. 2:**
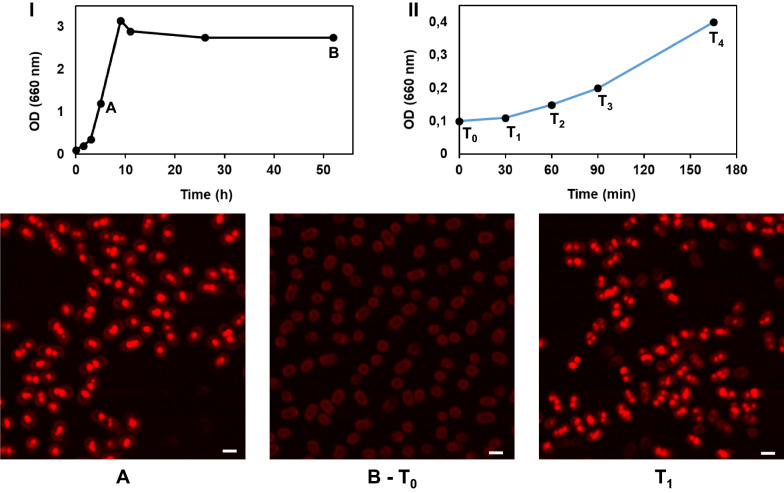
The PHB granules appear before the first cellular division. **A** Mid-exponential phase cells. **B** Late stationary phase cells, used as inoculum (T_0_) for the second time-series experiment. T_1_ Cells after 30 min of shaking at 34 °C and 300 rpm. The microscopy images of T2, T3 and T4 samples are not shown in Fig. 2 because they are very similar to the T1 sample. The scale bar is 1 µm

We then investigated the kinetics of PHB accumulation in *P. denitrificans* cells during growth on mineral salts medium with succinate. A time-series experiment was performed and samples were collected periodically for simultaneous determination of biomass, PHB content and microscopic performance. We noted that the specific PHB content per dry weight biomass remained constant at a level of about 7% during the exponential phase of growth. Once at the stationary phase, the PHB granules were completely consumed within less than 25 h, as judged by gas chromatography and microscopy analyses that show that these starved cells lack Nile Red fluorescent foci (see Additional file 1: Figure S2). We then used these starved cells (see sample B in I of Fig. 2) as inoculum for new cultures with fresh medium and succinate. These cultures were grown at 34 ºC and samples were collected at different time points along with the early exponential phase of growth (from 30 to 165 min; see samples T1–T4 in panel II of Fig. 2). Each sample was incubated with Nile Red for 5 min and imaged by LSCM to observe the presence and distribution of the PHB granules. Results are shown in Fig. 2 and they show the presence of PHB granules already at the first time point (T1), which was 30 min after the inoculation of the new cultures with the starved cells. Since the biomass did not increase in that period, we can conclude that PHB synthesis occurs before cellular division.

**Fig. 3:**
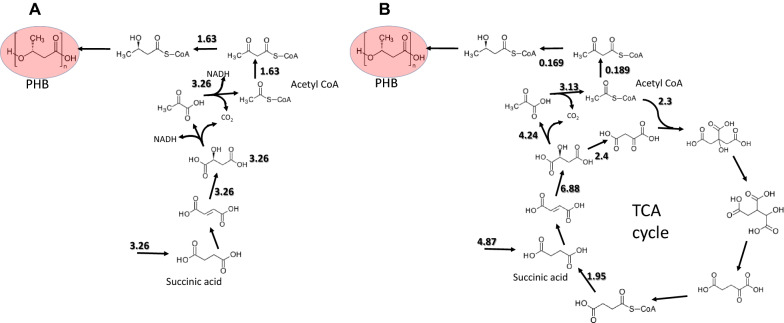
Distributions of metabolic fluxes under PHB accumulation and exponential growth. The numbers are reaction rates in mmol g-DW^−1^ h^−1^. Only the main metabolic fluxes are shown

### Modelling PHB synthesis in *P. denitrificans *cells growing on succinate as sole carbon and energy sources

To model PHB synthesis in *P. denitrificans,* we performed a third time series experiment using the same defined mineral salts medium with 25 mM succinate as the sole carbon and energy and carbon sources and a similar amount of inoculum (0.04 mg ml^−1^), but with a lower frequency of shaking (150 rpm; so as not to have increase in the number of cells in the first 30 min of culture). Experiments were carried out in duplicate and samples were collected periodically for simultaneous determination of dry weight of the biomass, PHB content and residual concentration of succinate in the medium. Data of these analyses are shown in Table 1.

**Table 1:** Table showing dry weight biomass, PHB content and residual succinate concentration in the medium

Time (h)	Dry weight biomass (mg ml^−1^)	PHB production per dry weight biomass (µg mg^−1^)	[Suc]_mM_
T_0_	0.04 ± 0.00	00.0 ± 0.0	25.0 ± 0.0
T_0.5_	0.04 ± 0.00	78.4 ± 8.4	24.9 ± 0.0
T_2_	0.06 ± 0.01	74.6 ± 9.1	23.7 ± 0.3
T_4_	0.07 ± 0.01	75.0 ± 8.3	23.2 ± 0.9
T_7_	0.21 ± 0.03	66.7 ± 2.7	20.3 ± 0.5
T_10_	0.59 ± 0.07	72.3 ± 1.4	15.7 ± 0.6
T_12_	0.82 ± 0.03	68.9 ± 5.4	9.1 ± 0.3
T_14_	1.01 ± 0.01	72.4 ± 5.4	4.7 ± 0.5
T_16_	1.12 ± 0.03	73.6 ± 9.3	0.9 ± 0.2

The slope of the logarithm of biomass during the exponential phase versus time showed a specific growth rate of 0.232 ± 0.002 h^−1^. The slope of the produced biomass versus the consumed succinate indicates a biomass yield on succinate of 48.4 ± 2.1 g-DW mol^−1^. Dividing the specific growth rate by the yield, we obtain a specific succinate uptake rate of 4.8 ± 0.2 mmol g-DW^−1^ h^−1^. Assuming a constant PHB content of 7% during the exponential phase, the specific rate of PHB production is equal to 16.2 mg PHB g-DW^−1^ h^−1^. With a molar mass of 96 g mol^−1^ (the mass of the monomer minus a water molecule lost during polymerization), the molar PHB production rate is 17 mmol g-DW^−1^ h^−1^. The experimentally measured specific growth rate and PHB production rates were set as constraints in a GSMM of *P. denitrificans*. Flux Balance Analysis (FBA) was carried out by minimizing the rate of succinate uptake rate. Maintenance ATP costs were calculated from the maintenance substrate uptake rates reported by van Verseveld et al. (1979) (the ordinate at the origin in Pirt’s equation). A value of 7.5 mmol ATP g-DW^−1^ h^−1^ for maintenance was obtained and was imposed as an extra constraint in the model. The FBA simulation predicted a specific succinate uptake rate of 4.87, which agrees almost perfectly with the experimental result, confirming the accuracy of the used GSMM. FBA simulations yield predictions of the reaction rates for every metabolic enzyme in the model.

The PHB content increased from zero to 7.8% in 30 min, which corresponds to a specific accumulation rate of 1.63 mmol g-DW^−1^ h^−1^. This rate is ten times higher than the one observed during exponential growth. In the absence of growth, and assuming the absence of CO_2_ fixation by the Calvin cycle, all the consumed succinate is transformed into acetyl-CoA as is shown in Fig. 3A. The rates of synthesis of acetyl-CoA predicted by the GSMM both during the initial PHB accumulation and during exponential growth are very close (3.26 versus 3.13 mmol g-DW^−1^ h^−1^). This suggests that during exponential growth the rate of PHB synthesis decreases drastically due to the fact that TCA cycle enzymes consume acetyl-CoA for biosynthetic pathways necessary to support growth (Fig. 3B). According to the model, the TCA cycle remains inactive during the initial phase of PHB accumulation, due to the fact that the NADH obtained in the reactions catalyzed by the malic enzyme (ME) and pyruvate dehydrogenase (PDH) is sufficient to feed the respiratory chain and generate the ATP necessary for cell maintenance. The production of PHB in *P. denitrificans* correlates well with acetyl-CoA levels, with higher levels leading to higher PHB production.

The metabolic flux distributions predicted by the GSMM also revealed the number of metabolic enzymes required for PHB accumulation and ATP production in each of the phases. According to this estimation, the cell needs to express only 50 metabolic enzymes in the first PHB accumulation phase, while during the exponential growth phase, 218 different metabolic enzymes are required (see the list of these enzymes in Additional file 2: File S1).

## Discussion

Here we show a sequential onset of PHB formation and remaining modes of cellular metabolism in *P. denitrificans* cells that were previously starved and lacked PHB granules. PHB granules were synthesized within 30 min of growth in fresh media with succinate reaching a percentage of 7%. This activity required only 50 metabolic enzymes as judged by our calculations. Metabolic routes essential for growth appeared to be silenced in this initial phase as judged by the fact that there was no increase in biomass. Only after the formation of the granules, did additional cellular metabolism occur, resulting in cell division and an exponential increase in cellular biomass. In this second phase, 218 enzymes are required as a minimum set of metabolic enzymes. PHB remained constant in this period at the initial level of 7%.

Kojima and co-workers (Kojima et al. 2004) showed that the production of PHB in *P. denitrificans* is linked to acetyl-CoA levels, with higher levels leading to higher PHB production. The same authors reported that PHB synthesis in *P. denitrificans* is not transcriptionally regulated. The expression of the genes *phaA*, *phaB* and *phaC* (Kojima et al. 2004), involved in PHB biosynthesis, is not repressed in presence of nitrogen, in contrast to other microorganisms, which only accumulate PHB when growth is arrested due to nitrogen limitation (Bordel et al. 2019a, b). Kojima et al. (2004) showed that the PHB biosynthetic genes were not repressed in presence of nitrogen, however, the PHB content of *P. denitrificans* cells decreased drastically when nitrogen was available. This drop of PHB was concomitant to a strong decrease in the cellular concentration of acetyl-CoA. These observations are consistent with our simulations. In conditions of exponential growth, most of the produced acetyl-CoA is drained into the TCA cycle, and, in lower amounts, to the synthesis of other metabolic precursors. This results in the depletion of the cellular pool of acetyl-CoA, which in turn causes a strong decrease in the rate of acetoacetyl-CoA synthesis by beta-ketothiolase (*phaA*). The same correlation between PHB accumulation and acetyl-CoA concentration was also observed by Olaya-Abril et al. (2018) in a mutant strain with increased PHB accumulation. The stoichiometry of beta-ketothiolase involving 2 acetyl-CoA molecules is likely to make its rate more sensitive to decreased acetyl-CoA concentrations (if the concentrations are below the enzyme saturation levels). For instance, a four-fold drop in the concentration of acetyl-CoA (as the one reported by Kojima and co-workers), would result in a 16-fold decrease of the rate of beta-ketothiolase, provided that the expression level of the enzyme remains the same. This fact could explain why citrate synthase outcompetes beta-ketothiolase under exponential growth conditions.

A good linear correlation between Nile Red staining and PHB content has been well established in many previous works using different bacteria (Gorenflo et al. 1999; Spiekermann et al. 1999; Zuriani et al. 2013). Our results are similar to the results of different fluorescence microscopy studies on PHB granule formation. For example, when PHB-free cultures of *Azotobacter vinelandii* or *Caryophanon latum* were transferred to fresh media with suitable carbon and energy sources, formation of visible fluorescent Nile Red stained structures appeared after 1 or 2 h of incubation (Hermawan and Jendrossek 2007; Jendrossek et al. 2007). PHB accumulation was even fastest in *R. eutropha* cells, which needed only 10 min to accumulate PHB granules after transfer of PHB-free cells to a medium promoting PHB accumulation (Jendrossek 2009). This is of the same order of time in *P. denitrificans* cells.

Models in the literature for granule formation suggest that granules may arise through physical association of PHB oligomers forming micelle-like structures or by budding off the plasma membrane, resulting in a granule covered with a monolayer of lipid (Jendrossek 2009). It has been observed that the distribution of PHB granules in *Rhodospirillum rubrum* and *R. eutropha* occurred near the cell poles and near the cell wall (Jendrossek 2009). Indeed, we occasionally observed a more regular distribution of the PHB granules in the *P. denitrificans* cells. We hypothesize that an equal distribution of PHB granules is required in dividing cells perhaps by using a division control mechanism of the PHB inclusions explaining the more organized distribution of the granules. Interestingly, *R. rubrum* cells exhibited a regular PHB granule distribution pattern as well with almost equal spacing between individual granules (Handrick et al. 2004), similar to what we observed in this study. In the literature, there are contradictory conclusions about the localization of the nascent PHB granules within the cell. In some cases, the granules appear dispersed randomly in the cytoplasm and in other cases, the nascent granules are shown to be attached to the inner leaflet of the plasma membrane (Tian et al. 2005). Future studies are required to get a more fundamental understanding of the organization of PHB granules in different species and at different phases of growth.

## Supplementary Information

**Additional file 1:Figure S1.** Staining of *P. denitrificans* cells with MTG. Details of dividing cells (at an OD660 of about 0.75) (**A**) and spheroplasts (**B**) stained with 1μM MTG during 5 min at 34 ºC and imaged using LSCM showing PHB granules distributed symmetrically. **Figure S2.** PHB inclusions in *P. denitrificans* cells grown in a defined mineral salts medium with 25 mM succinate as the sole carbon and energy sources. **A** Growth curve of the cells incubated at 34°C and 300 rpm and gas chromatographic data of specific PHB production (μg PHB per mg of cell biomass). **B** At different time points, 1ml of culture was incubated with 2 μM of Nile Red during 5 min and imaged using LSCM. The images show the disappearance of PHB inclusions at the stationary phase of growth and completely disappeared after 34 h of cultivation.

**Additional file 2:File S1.** Excel with the list of these enzymes mentioned in the text.

## Data Availability

The authors declare that all data obtained have been included into the manuscript, its Additional file 1, 2 and/or repositories.

## References

[CR1] Anderson AJ, Dawes EA (1990). Occurrence, metabolism, metabolic role, and industrial uses of bacterial polyhydroxyalkanoates. Microbiol Rev.

[CR2] Bordel S, Rojas A, Muñoz R (2019). Reconstruction of a Genome Scale Metabolic Model of the polyhydroxybutyrate producing methanotroph *Methylocystis parvus* OBBP. Microb Cell Fact.

[CR3] Bordel S, Rodríguez Y, Hakobyan A, Rodríguez E, Lebrero R, Muñoz R (2019). Genome scale metabolic modeling reveals the metabolic potential of three type II methanotrophs of the genus Methylocystis. Metab Eng.

[CR4] Dariš B, Knez Ž (2020). Poly(3-hydroxybutyrate): Promising biomaterial for bone tissue engineering. Acta Pharm.

[CR5] De Vries GE, Harms N, Hoogendijk J, Stouthamer AH (1989). Isolation and characterization of *Paracoccus denitrificans* mutants with increased conjugation frequencies and pleiotropic loss of a (nGATCn)-DNA-modifying property. Arch Microbiol.

[CR6] Ebrahim A, Lerman JA, Palsson BO, Hyduke DR (2013). COBRApy: constraints-based reconstruction and analysis for python. BMC Syst Biol.

[CR7] Gorenflo V, Steinbüchel A, Marose S, Rieseberg M, Scheper T (1999). Quantification of bacterial polyhydroxyalkanoic acids by Nile red staining. Appl Microbiol Biotechnol.

[CR8] Handrick R, Reinhardt S, Schultheiss D, Reichart T, Schüler D, Jendrossek V, Jendrossek D (2004). Unraveling the function of the *Rhodospirillum rubrum* activator of polyhydroxybutyrate (PHB) degradation: the activator is a PHB-granule-bound protein (phasin). J Bacteriol.

[CR9] Harms N, De Vries GE, Maurer K, Veltkamp E, Stouthamer AH (1985). Isolation and characterization of *Paracoccus denitrificans* mutants with defects in the metabolism of one-carbon compounds. J Bacteriol.

[CR10] Hermawan S, Jendrossek D (2007). Microscopical investigation of poly(3-hydroxybutyrate) granule formation in *Azotobacter vinelandii*. FEMS Microbiol Lett.

[CR11] Jendrossek D (2005). Fluorescence microscopic investigation of poly(3-hydroxybutyrate) granule formation in bacteria. Biomacromol.

[CR12] Jendrossek D (2009). Polyhydroxyalkanoate granules are complex subcellular organelles (carbonosomes). J Bacteriol.

[CR13] Jendrossek D, Handrick R (2002). Microbial degradation of polyhydroxyalkanoates. Annu Rev Microbiol.

[CR14] Jendrossek D, Pfeiffer D (2014). New insights in the formation of polyhydroxyalkanoate granules (carbonosomes) and novel functions of poly(3-hydroxybutyrate). Environ Microbiol.

[CR15] Jendrossek D, Selchow O, Hoppert M (2007). Poly(3-hydroxybutyrate) granules at the early stages of formation are localized close to the cytoplasmic membrane in *Caryophanon latum*. Appl Environ Microbiol.

[CR16] Kojima T, Nishiyama T, Maehara A, Ueda S, Nakano H, Yamane T (2004). Expression profiles of polyhydroxyalkanoate synthesis-related genes in *Paracoccus denitrificans*. J Biosci Bioeng.

[CR17] Olaya-Abril A, Luque-Almagro VM, Manso I, Gates AJ, Moreno-Vivián C, Richardson DJ, Roldán MD (2018). Poly(3-hydroxybutyrate) hyperproduction by a global nitrogen regulator NtrB mutant strain of *Paracoccus denitrificans* Pd1222. FEMS Microbiol Lett.

[CR18] Overbeek R, Begley T, Butler RM, Choudhuri JV, Chuang HY, Cohoon M, de Crécy-Lagard V, Diaz N, Disz T, Edwards R, Fonstein M, Frank ED, Gerdes S, Glass EM, Goesmann A, Hanson A, Iwata-Reuyl D, Jensen R, Jamshidi N, Krause L, Kubal M, Larsen N, Linke B, McHardy AC, Meyer F, Neuweger H, Olsen G, Olson R, Osterman A, Portnoy V, Pusch GD, Rodionov DA, Rückert C, Steiner J, Stevens R, Thiele I, Vassieva O, Ye Y, Zagnitko O, Vonstein V (2005). The subsystems approach to genome annotation and its use in the project to annotate 1000 genomes. Nucleic Acids Res.

[CR19] Overbeek R, Olson R, Pusch GD, Olsen GJ, Davis JJ, Disz T, Edwards RA, Gerdes S, Parrello B, Shukla M, Vonstein V, Wattam AR, Xia F, Stevens R (2014). The SEED and the rapid annotation of microbial genomes using subsystems technology (RAST). Nucleic Acids Res.

[CR20] Potter M, Steinbüchel A (2005). Poly(3-hydroxybutyrate) granule-associated proteins: impacts on poly(3-hydroxybutyrate) synthesis and degradation. Biomacromol.

[CR21] Reinecke F, Steinbüchel A (2009). *Ralstonia eutropha* strain H16 as model organism for PHA metabolism and for biotechnological production of technically interesting biopolymers. J Mol Microbiol Biotechnol.

[CR22] Spiekermann P, Rehm BH, Kalscheuer R, Baumeister D, Steinbüchel A (1999). A sensitive, viable-colony staining method using Nile red for direct screening of bacteria that accumulate polyhydroxyalkanoic acids and other lipid storage compounds. Arch Microbiol.

[CR23] Steinbüchel A, Aerts K, Babel W, Follner C, Liebergesell M, Madkour MH, Mayer F, Pieper-Furst U, Pries A, Valentin HE (1995). Considerations on the structure and biochemistry of bacterial polyhydroxyalkanoic acid inclusions. Can J Microbiol.

[CR24] Tian J, Sinskey AJ, Stubbe J (2005). Kinetic studies of polyhydroxybutyrate granule formation in *Wautersia eutropha* H16 by transmission electron microscopy. J Bacteriol.

[CR25] Ueda S, Matsumoto S, Takagi A, Yamane T (1992). Synthesis of poly(3-hydroxybutyrate-co-3-hydroxyvalerate) from methanol and n-amyl alcohol by the methylotrophic bacteria *Paracoccus denitrificans* and *Methylobacterium extorquens*. Appl Environ Microbiol.

[CR26] Van Verseveld HW, Boon JP, Stouthamer AH (1979). Growth yields and the efficiency of oxidative phosphorylation of *Paracoccus denitrificans* during two-(carbon) substrate-limited growth. Arch Microbiol.

[CR27] Vida TA, Emr SD (1995). A new vital stain for visualizing vacuolar membrane dynamics and endocytosis in yeast. J Cell Biol.

[CR28] Zúñiga C, Morales M, Le Borgne S, Revah S (2011). Production of poly-β-hydroxybutyrate (PHB) by *Methylobacterium organophilum* isolated from a methanotrophic consortium in a two-phase partition bioreactor. J Hazard Mater.

[CR29] Zuriani R, Vigneswari S, Azizan MNM, Majid MIA, Amirul AA (2013). A high throughput Nile red fluorescence method for rapid quantification of intracellular bacterial polyhydroxyalkanoates. Biotechnol Bioprocess Eng.

