# Supplementary material for: Imaging and modelling of poly(3-hydroxybutyrate) synthesis in Paracoccus denitrificans
Source: AMB Express. 2021 Aug 9;11:113. doi: 10.1186/s13568-021-01273-x (PMC8353029; doi:10.1186/s13568-021-01273-x)
Supplement: Supplementary file 1 — Additional file 1:Figure S1. Staining of P. denitrificans cells with MTG. Details of dividing cells (at an OD660 of about 0.75) (A) and spheroplasts (B) stained with 1μM MTG during 5 min at 34 ºC and imaged using LSCM showing PHB granules distributed symmetrically. Figure S2. PHB inclusions in P. denitrificans cells grown in a defined mineral salts medium with 25 mM succinate as the sole carbon and energy sources. A Growth curve of the cells incubated at 34°C and 300 rpm and gas chromatographic data of specific PHB production (μg PHB per mg of cell biomass). B At different time points, 1ml of culture was incubated with 2 μM of Nile Red during 5 min and imaged using LSCM. The images show the disappearance of PHB inclusions at the stationary phase of growth and completely disappeared after 34 h of cultivation. [file 13568_2021_1273_MOESM1_ESM.pdf]

SUPPLEMENTARY MATERIAL

AMB Express

Imaging and modelling of poly(3-hydroxybutyrate) synthesis in *Paracoccus denitrificans*

Sergio Bordel[1,2], Rob J. M. van Spanning[3], Fernando Santos-Beneit*[1,2,3]

1. Department of Chemical Engineering and Environmental Technology, School of Industrial Engineering, University of Valladolid, Dr. Mergelina, s/n, 47011 Valladolid, Spain

2. Institute of Sustainable Processes, Dr. Mergelina s/n, 47011 Valladolid, Spain

3. Department of Molecular Cell Biology, Faculty of Science, Vrije Universiteit Amsterdam, The Netherlands

* fernando.santos.beneit@uva.es

Fernando Santos-Beneit: https://orcid.org/0000-0001-7986-5557

Sergio Bordel: https://orcid.org/0000-0001-6162-6478

Supplementary File S1. Excel with the list of these enzymes mentioned in the text.

Supplementary Figure S1. Staining of *P. denitrificans* cells with MTG. Details of dividing cells (at an OD660 of about 0.75) (A) and spheroplasts (B) stained with 1µM MTG during 20 min at 34ºC and imaged using LSCM showing PHB granules distributed symmetrically.

Supplementary Figure S2. PHB inclusions in *P. denitrificans* cells grown in a defined mineral salts medium with 25 mM succinate as the sole carbon and energy sources. A) Growth curve of the cells incubated at 34°C and 300 rpm and gas chromatographic data of specific PHB production (µg PHB per mg of cell biomass). B) At different time points, 1ml of culture was incubated with 2 µM of Nile Red during 5 min and imaged using LSCM. The images show the disappearance of PHB inclusions at the stationary phase of growth and completely disappeared after 34 hours of cultivation.

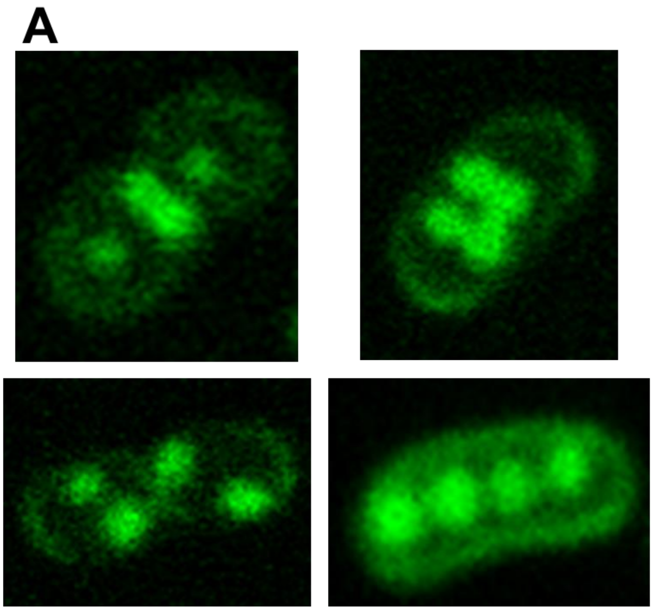

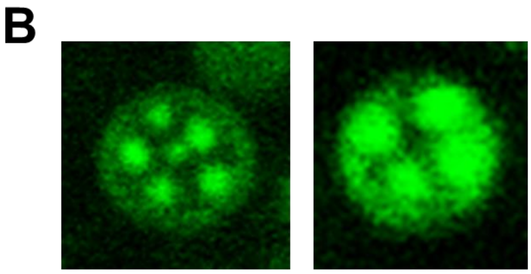

Figure S1: Staining of *P. denitrificans* cells with MTG. Details of dividing cells (at an OD660 of about 0.75) (A) and spheroplasts (B) stained with 1µM MTG during 20 min at 34ºC and imaged using LSCM showing PHB granules distributed symmetrically.

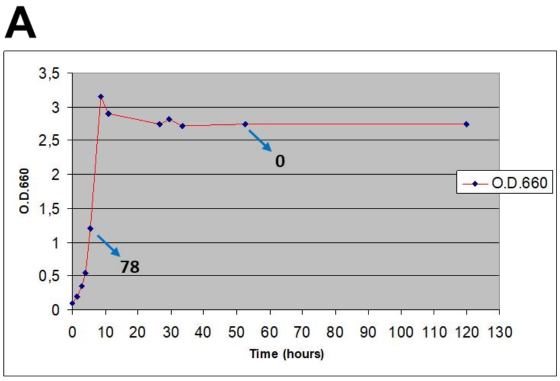

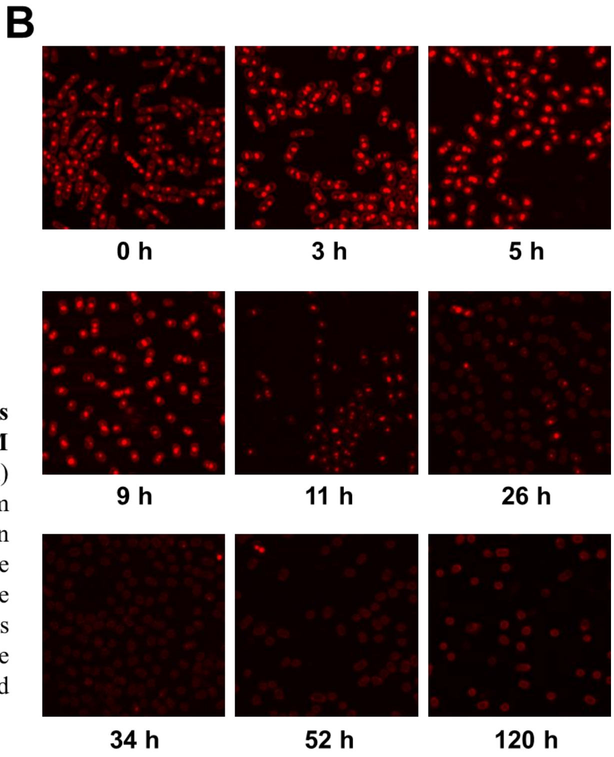

Figure S2: PHB inclusions in P. denitrificans cells grown in a defined mineral salts medium with 25 mM succinate as the sole carbon and energy sources. A) Growth curve of the cells incubated at 34°C and 300 rpm and gas chromatographic data of specific PHB production (µg PHB per mg of cell biomass). B) At different time points, 1ml of culture was incubated with 2 µM of Nile Red during 5 min and imaged using LSCM. The images show the disappearance of PHB inclusions at the stationary phase of growth and completely disappeared after 34 hours of cultivation.
